# Five-Year Trends in Dietary Intake by Gender Among Korean Preschoolers (2017–2021): Nationwide Survey-Based Surveillance Amid Rising Demand for Medical Care for Precocious Puberty

**DOI:** 10.3390/nu18203445

**Published:** 2026-10-20

**Authors:** Junho Yang, Jinyoung Lee, Hyunji Og, Minsoo Jeong, Seungjun Lee

**Affiliations:** 1 Institute for Agricultural Product Quality & Safety Assessment, Kyungpook National University, Daegu 41566, Republic of Korea; jh.yang@knu.ac.kr; 2 Department of Food Sciences and Nutrition, Pukyong National University, Busan 48513, Republic of Korea; jinsgod83@gmail.com (J.L.); okhj0814@pukyong.ac.kr (H.O.); 3 NGS Center, Kyungpook National University, Daegu 41566, Republic of Korea; minsoo0326@knu.ac.kr; 4 Department of Applied Biosciences, Kyungpook National University, Daegu 41566, Republic of Korea

**Keywords:** precocious puberty, sex-specific differences, dietary intake trends, nutritional surveillance, Korean children

## Abstract

Background/Objectives: Care-seeking for precocious puberty (PP) in South Korea has risen sharply, coinciding with dietary westernization among children. This study describes sex-specific and temporal dietary trends among Korean children aged 1–11 years, focusing on the 6–11-year band preceding the typical PP diagnosis threshold. Methods: Using KNHANES (2017–2021) dietary data and HIRA care-seeking counts with the sex-year aggregate, not the individual child. Sex differences were assessed using the Mann–Whitney U test, year-to-year patterns using a sex-by-year interaction model (*n* = 5 annual observations per sex), and dietary pattern structure using principal component analysis (PCA) on 12 food groups (*n* = 10 sex-year observations). Results: PP care-seeking rates rose in both sexes, substantially more in girls. Boys showed significantly higher intake of most food groups and macronutrients; within the 6–11-year band this held for meat, cola, and macronutrients (*p* = 0.008) but not legumes (*p* = 0.421). Macronutrient sex gaps numerically narrowed while meat and cola gaps widened over 2017–2021, though none reached significance. PCA identified a primary axis reflecting overall intake volume that separated boys and girls, and a secondary axis distinguishing a dairy/seaweed/beverage profile from a meat/egg/cola-dominant profile. Conclusions.: These findings describe sex-specific dietary patterns amid rising PP rates without establishing that intake volume explains the sex disparity, identifying candidate exposures for future research and cautioning against oversimplified dietary narratives.

## 1. Introduction

Precocious puberty is defined as the development of secondary sexual characteristics at an earlier age than typically expected for a child’s sex [1,2]. Clinically, it is diagnosed when breast development begins before age 8 in girls and when testicular enlargement occurs before 9 years in boys [1,2]. Pubertal maturation normally follows a tightly regulated sequence governed by complex neuroendocrine mechanisms. Precocious puberty is clinically classified into two subtypes: central precocious puberty, which arises from the premature activation of the hypothalamic-pituitary-gonadal (HPG) axis and accounts for the large majority of cases, particularly in girls [3,4]; and peripheral precocious puberty, which results from autonomous sex-hormone production independent of HPG-axis activation (e.g., gonadal or adrenal tumors, or exogenous hormone exposure) and is comparatively rare. The mechanistic discussion in this manuscript, including the metabolic pathways considered in the Discussion, pertains specifically to central precocious puberty. Early activation of this axis potentially leads to accelerated bone maturation, premature epiphyseal closure, psychosocial distress, and an increased risk of long-term metabolic disorders [5,6]. The prevalence of precocious puberty exhibits a marked sex discrepancy, occurring approximately ten times more frequently in girls than in boys [7,8], reflecting substantial sex-specific vulnerability in pubertal timing.

This phenomenon can be interpreted within the context of a global trend toward earlier pubertal onset. In recent decades, the incidence of central precocious puberty has steadily increased worldwide [9]. Notably, South Korea has experienced a disproportionate rise in precocious puberty compared to other developed nations. In 2012, the Korean government designated precocious puberty as a “disease of national concern,” and in 2018, it was included in the list of the “top 100 diseases closely related to daily life and of high public interest” [10]. Epidemiologic data reveal a sharp increase in incidence, from 15.9 per 100,000 between 2004 and 2010 to 122.8 per 100,000 based on analyses of 2008–2014 data [11]. Improvements in living standards and nutritional status, along with rapid lifestyle changes, presumably contribute to this trend, which intensified notably during the COVID-19 pandemic [12,13]. Increasing childhood obesity and prolonged mobile device use have been identified as key factors associated with the earlier onset and accelerated progression of puberty [14,15]. Consequently, the number of children undergoing clinical evaluations for suspected precocious puberty has increased substantially. While girls typically present earlier and progress more rapidly, boys—though less frequently affected—are more likely to have identifiable organic causes, necessitating thorough diagnostic assessments [16].

Beyond general lifestyle modifications, growing interest lies in the role of diet in influencing pubertal timing. A few epidemiologic studies have suggested that, in addition to energy imbalance, dietary intake is significantly associated with the timing of puberty, with childhood obesity—driven by a Westernized diet high in meat, saturated fats, and processed foods, and low in dietary fiber—identified as a major risk factor for early pubertal development [17]. High consumption of animal-based protein, particularly red meat, has been linked to an increased risk of precocious puberty, potentially advancing puberty onset by up to 7 months [18,19]. Similarly, regular intake of sugar-sweetened beverages is associated with an elevated risk of early pubertal development [18]. These findings indicate that childhood diet is a modifiable factor that influences pubertal development, suggesting that both the timing of puberty and related environmental exposures may be responsive to preventive interventions. Note that these epidemiologic studies of dietary influences on puberty typically measure early pubertal timing as a population-level statistical shift (e.g., an earlier average age at breast onset or menarche), which is conceptually distinct from precocious puberty as a clinical diagnosis defined by fixed age thresholds; the two concepts are related but not interchangeable.

Despite heightened interest in dietary influences, comprehensive population-level analyses that integrate national dietary intake data with disease incidence are limited. Elucidating the relationship between specific nutrient or food group consumption patterns and trends in precocious puberty incidence is essential for informing evidence-based prevention strategies and avoiding oversimplified dietary narratives. Rather than testing whether population-level dietary intake explains the sex disparity in PP care-seeking—a comparison that would be confounded by the substantial sex differences in pubertal timing and hormonal regulation described above—this study aimed to (1) characterize sex-specific and year-to-year trends in the intake of food groups and macronutrients showing a significant overall sex difference (meat, legumes, cola, carbohydrate, protein, animal protein, and fat) among children aged 1–11 years between 2017 and 2021, with particular attention to the 6–11-year band—the age range immediately preceding the typical clinical threshold for PP diagnosis—and (2) examine the underlying structure of dietary patterns among these food groups and macronutrients using multivariate analysis. National PP care-seeking trends over the same period, likewise, examined by age band, are presented as contextual background motivating this dietary surveillance, rather than as an outcome explained by the intake data. We selected this band because it is the age-grouping convention common to both KNHANES and HIRA, not because it is intended to align precisely with the sex-specific clinical age thresholds for PP (before 8 years in girls, before 9 years in boys), which fall within this band rather than at its boundary and differ slightly by sex.

To this end, dietary intake data from the 2017–2021 Korea National Health and Nutrition Examination Survey (KNHANES) were analyzed, and national PP care-seeking counts from the Health Insurance Review and Assessment Service (HIRA) are reported separately to establish the public health context for this survey. This approach seeks to provide a transparent, appropriately scoped description of Korean children’s dietary trends, offering candidate exposures for future individual-level research rather than premature causal claims regarding diet-related risk factors in early pubertal development. This study is best understood as a sex-specific, nationally representative dietary surveillance analysis—particularly within the 6–11-year band—with national PP-related care-seeking trends presented alongside it as descriptive background information. Because the two datasets are not linked to one another, this study design has limited capacity to identify novel dietary determinants of precocious puberty.

## 2. Materials and Methods

### 2.1. Data Collection

To examine sex-specific and temporal patterns in the dietary intake of young Korean children during a period of rising national precocious puberty (PP) care-seeking rates, we compiled dietary intake data from a national nutrition survey together with national PP care-seeking counts reported separately as background context. Both data sources were stratified into three age bands common to KNHANES and HIRA age groupings (1–2, 3–5, and 6–11 years); children aged 12 years and older were excluded, as PP care-seeking counts in this range approach zero and are uninformative for this analysis. All dietary analyses were conducted at the sex-year aggregate with five years of observation per sex rather than focusing on individual patients, and no individual-level linkage between dietary intake and PP care-seeking was attempted or implied.

#### 2.1.1. Precocious Puberty Care-Seeking Counts (Background Context)

Precocious puberty is generally defined as the onset of puberty before age 8 in girls and before age 9 in boys [20]. National HIRA claims data for children aged 1–11 years (2017–2021), stratified by the same age bands as the dietary data, record the number of individuals who received care coded under disease classification code E301 in each calendar year [11]. A total of 582,183 such cases were recorded across this population during the study period. Because these counts capture all care coded under E301—including diagnostic work-up and follow-up visits rather than only newly confirmed diagnoses—age-band-specific rates derived from them (cases per 100,000 population) are reported here as a descriptive measure of relative care-seeking across age bands, sexes, and years, rather than as directly comparable to published incidence estimates for confirmed new PP diagnoses [11]. Additionally, code E301 does not itself distinguish central from peripheral precocious puberty, so these care-seeking counts likely reflect a mixture of both subtypes, though central precocious puberty is clinically the more common subtype, particularly among girls; these counts are not used as an outcome variable in the statistical analyses below.

#### 2.1.2. KNHANES Dietary Intake Data

This study utilized dietary intake data from the KNHANES, conducted by the Korea Disease Control and Prevention Agency (KDCA) during its seventh and eighth cycles (2017–2021) [21]. The KNHANES is a nationwide cross-sectional survey that employs a multi-stage clustered probability design to monitor the health and nutritional status of the South Korean population. For this study, we extracted dietary intake records for children aged 1–11 years to analyze population-level nutritional patterns.

The dataset encompasses daily average intake values categorized into 12 distinct food groups plus four macronutrients (carbohydrate, protein, animal protein, and fat), reported by sex, year, and age band (1–2, 3–5, and 6–11 years). These publicly released estimates reflect KNHANES’s complex sampling design (survey weights, strata, and clustering) as calculated by KDCA; the standard errors shown in are the corresponding survey-weighted standard errors of each annual estimate, not measures of variability across individual participants. Each such estimate is derived from the sum of measured intake among analysis subjects aged 2 years and older within the relevant stratum, and for certain published indicators, KDCA additionally applies age standardization using the 2005 Korean population as the reference. However, the specific number of analysis subjects underlying each year-sex-age-band cell used in this study was not recorded when these publicly released aggregate estimates were originally compiled, and is not available to us; we identify obtaining and reporting these sample sizes as an important addition for future work using these data.

The KNHANES endeavors to generate nationally representative and reliable statistics on health status, health behaviors, and dietary intake, serving as a foundational resource for formulating and evaluating public health goals, health promotion programs, and national health policies [22]. In the KNHANES food-group classification used here, cider refers to a non-alcoholic, carbonated soft drink, distinct from alcoholic cider; cola and carbonated drinks are tracked as mutually exclusive categories; and animal protein is reported as a nutritional subset of total protein, derived from animal-source foods.

### 2.2. Statistical Analysis

Statistical analyses included univariate, trend, and multivariate approaches. Univariate analyses were conducted using SPSS software (version 27; SPSS Inc., Chicago, IL, USA) for the Mann–Whitney U test, applied both to the full 1–11-year study population and, for the seven food groups/macronutrients with a significant overall sex difference, to the 6–11-year band specifically. Year-to-year sex differences in intake within the 6–11-year band were additionally evaluated using a sex-by-year weighted least-squares interaction model (weighted by the inverse variance of each annual survey estimate; Python 3.12, statsmodels), testing whether the slope of intake over time differed by sex (*n* = 5 annual observations per sex). Multivariate analyses of the 12 food groups (ages 6–11 years) were performed using SIMCA^®^ (version 18.2; Sartorius, Umeå, Sweden) for principal component analysis (PCA) with biplots, an approach previously applied to derive dietary patterns in pediatric nutrition surveillance [23,24]; the correlation structure between food-group intake and the four macronutrients was characterized separately using Pearson correlation with hierarchical clustering (average linkage, Euclidean distance; Python 3.12, SciPy).

#### 2.2.1. Mann–Whitney U Test

The Mann–Whitney U test is a non-parametric statistical method used to compare two independent groups when normality cannot be assumed. It determines whether one group tends to exhibit higher or lower values than another by ranking all observations and evaluating the sum of ranks for each group [25]. This test was utilized to compare the average daily intake of each food group between boys and girls over the study period (2017–2021), as a descriptive characterization of sex differences in children’s dietary intake, both overall (ages 1–11 years) and within the 6–11-year band specifically. Sex differences across all 12 food groups and four macronutrients were first screened using this test; the seven food groups and macronutrients showing a statistically significant overall sex difference—meat, legumes, cola, carbohydrate, protein, animal protein, and fat—were selected for the year-to-year trend analysis and re-examined within the 6–11-year band specifically. The five annual sex-specific estimates in each comparison are drawn from the same national survey years and are therefore not fully independent in the strict sense assumed by the Mann–Whitney test (e.g., shared survey-cycle and secular conditions across boys and girls within a given year), and each comparison uses only five observations per sex. To account for multiple comparisons across the 16 food groups and macronutrients screened in the overall comparison, we additionally applied a Benjamini–Hochberg false discovery rate (FDR) correction (q = 0.05); full results are reported in Supplementary Table S3. This selection was based on first-stage statistical significance in the pooled comparison rather than a priori biological hypotheses.

#### 2.2.2. Dietary Pattern Analysis (PCA)

Principal component analysis (PCA) with biplots was employed to explore the multivariate structure of dietary intake among the 12 KNHANES food groups, restricted to children aged 6–11 years and stratified by sex and year (2017–2021; 10 sex-year observations)—the age band immediately preceding the typical clinical threshold for PP diagnosis. The PCA input matrix was restricted to food-group intake variables; the four macronutrients (carbohydrate, protein, animal protein, fat), national PP care-seeking counts, and population denominators were not included as PCA variables, so that the resulting dietary pattern structure reflects co-variation among food groups independent of nutrient totals and PP care-seeking rates. Because macronutrient totals are derived in part from the food groups themselves (e.g., meat contributes directly to animal protein and fat), including both in a single PCA risks double-counting a shared signal; the relationship between the food-group pattern and macronutrient intake was therefore examined separately, using Pearson correlation between each food group and each macronutrient across the same 10 sex-year observations, with food groups and macronutrients each ordered by hierarchical clustering (average linkage, Euclidean distance on the correlation profile). Any parallel between the derived dietary pattern trends and national PP care-seeking trends is addressed separately and descriptively in the Discussion. To assess sensitivity to individual survey years, we repeated the PCA five times, each time excluding one year (both sexes, *n* = 8 remaining). PC1 loadings were highly correlated with the full-model loadings across all iterations (r = 0.61–0.97), and boys and girls remained cleanly separated on PC1 in every iteration. PC2 loadings were less stable (r = 0.33–0.95 across iterations), particularly when 2020 was excluded (r = 0.33), indicating that the secondary axis should be interpreted with more caution than the primary sex-separation axis. Because the number of variables (12 food groups) exceeds the number of observations (10 sex-year values), this PCA should be considered exploratory: prior to model fitting, each food-group variable was mean-centered and scaled to unit variance (UV-scaling/autoscaling), a standard preprocessing step for food-intake variables of differing units and magnitude; the resulting component structure cannot be assumed stable in the classical sense and is reported as a hypothesis-generating description of covariation among food groups rather than as a validated, replicable dietary pattern.

## 3. Results

### 3.1. Study Context: National Trends in Precocious Puberty Care-Seeking

**Figure 1:**
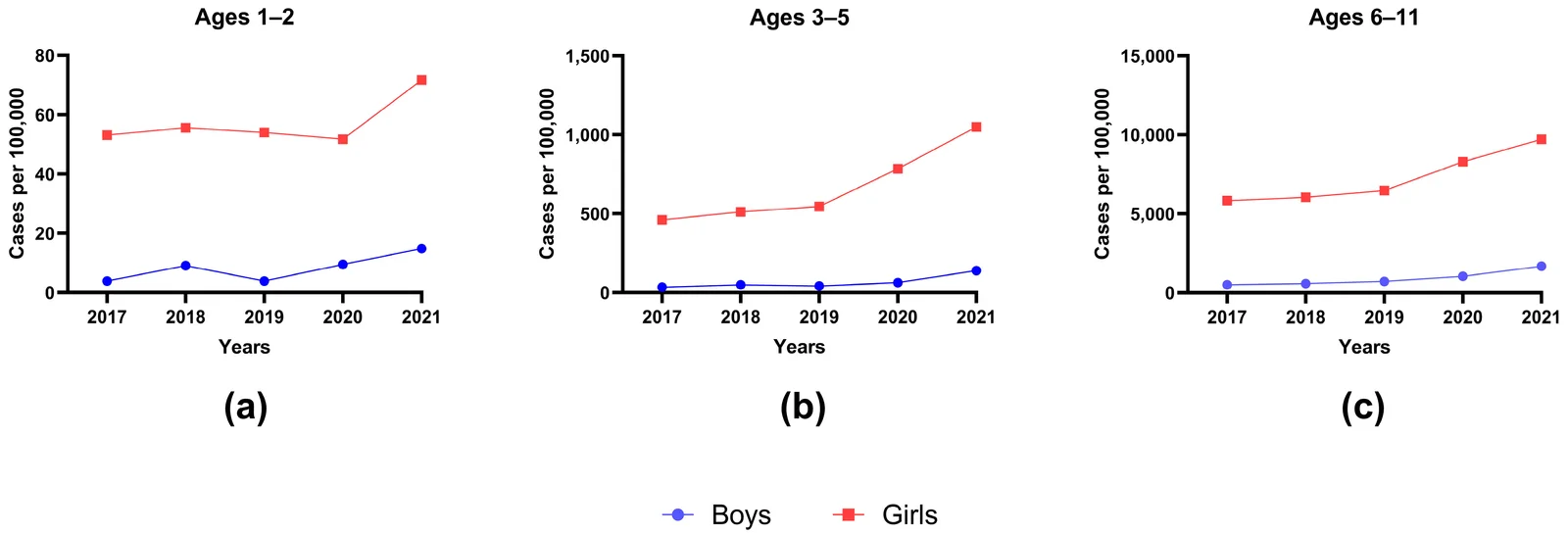
Age-band-specific national precocious puberty (PP) care-seeking rate (cases per 100,000 population), by sex, 2017–2021 (*n* = 5 year-points per sex per age band). (**a**) Ages 1–2 years; (**b**) ages 3–5 years; (**c**) ages 6–11 years.

As background motivating this dietary survey, Figure 1 presents HIRA-coded PP care-seeking rates (cases per 100,000 population, disease code E301; see Section 2.1.1.) in South Korea from 2017 to 2021, stratified by the same three age bands as the dietary data. Across the 1–11-year study population, the average number of cases per year was 13,384 ± 6917 among boys and 103,053 ± 23,027 among girls, with a persistent and marked sex disparity in every age band. This disparity, and its year-on-year increase, was most pronounced within the 6–11-year band, where the rate rose from 509 to 1676 per 100,000 among boys and from 5825 to 9723 per 100,000 among girls between 2017 and 2021 (Figure 1)—the age range immediately preceding the typical clinical threshold for PP diagnosis. Because HIRA claims counts reflect all care coded under E301 rather than confirmed new diagnoses specifically, these rates are substantially higher than, and not directly comparable to, previously published incidence estimates for confirmed PP (e.g., 15.9 to 122.8 per 100,000 [11]); they are presented here for relative comparison across age bands, sexes, and years within this dataset. These national trends are presented here solely to establish the public health relevance of the dietary patterns described below and are not treated as an outcome explained by the dietary data in this study.

### 3.2. Sex-Specific Dietary Intake Patterns (2017–2021)

Table 1 presents median daily intake across 12 food groups and four macronutrients for boys and girls, pooled across the five-year study period. Among food groups, dairy and meat intake were highest in both sexes, followed by fruit drinks and eggs; legumes, seafood, and seaweed contributed comparatively less, and sweetened/carbonated beverages were consumed in the smallest amounts. Among macronutrients, carbohydrate intake was highest, followed by protein, animal protein, and fat—values that are not independent of the food-group intakes reported alongside them, since macronutrient totals are derived in part from the same foods (Section 2.2.2). Overall, food-group and macronutrient intake patterns were broadly similar between boys and girls, with dairy, meat, and carbohydrate representing the largest contributors in both.

### 3.3. Age-Band-Specific Trends in Selected Food Groups and Macronutrients (Ages 6–11 Years), by Sex

**Figure 2:**
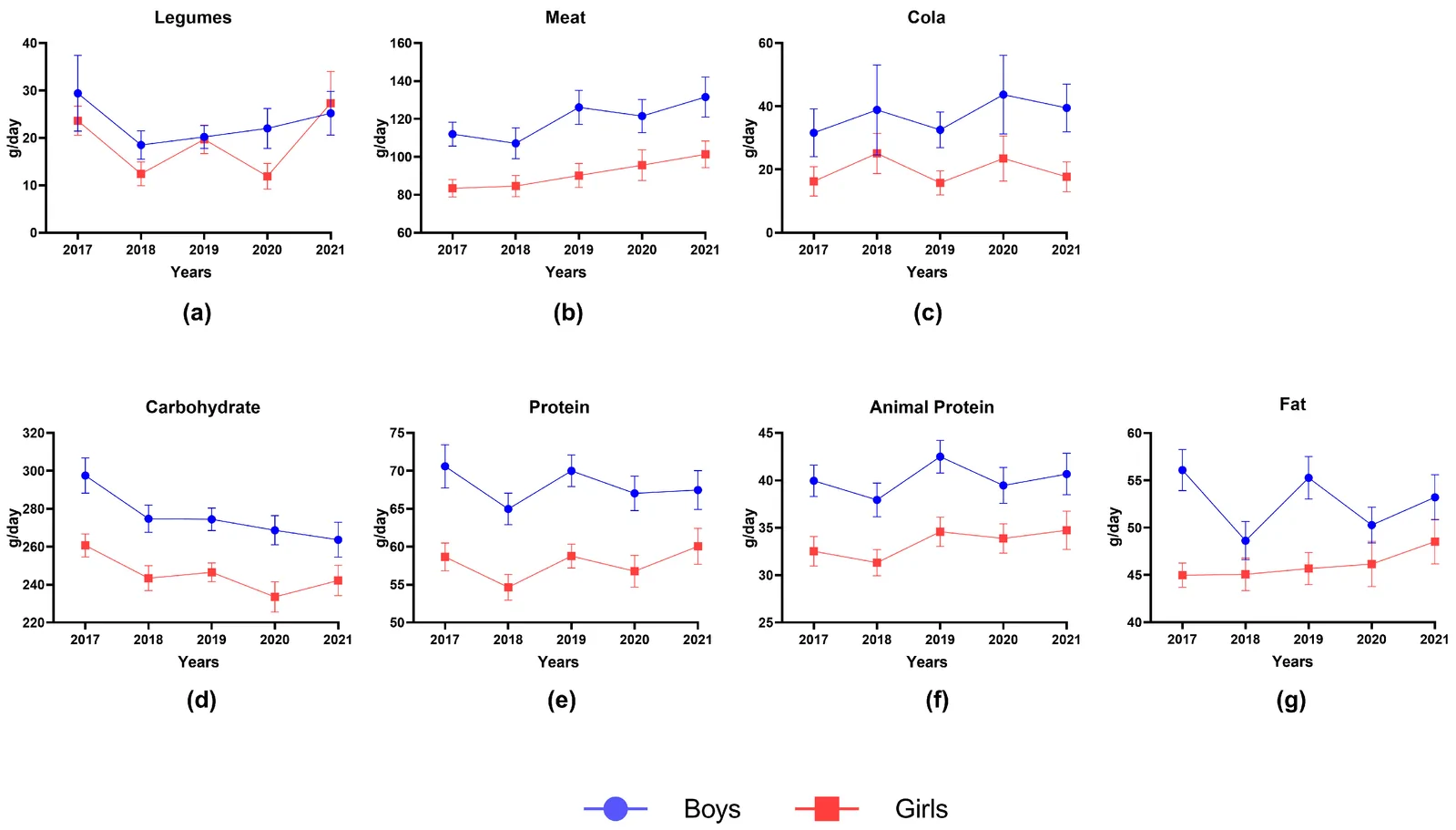
Year-to-year trends (2017–2021) in daily intake of the seven food groups/macronutrients with a significant overall sex difference, restricted to children aged 6–11 years, by sex (*n* = 5 year-points per sex). (**a**) Legumes; (**b**) meat; (**c**) cola; (**d**) carbohydrate; (**e**) protein; (**f**) animal protein; (**g**) fat. Data are shown as mean ± standard error of the annual survey estimate; the standard errors shown in Figure 2 are the corresponding survey-weighted standard errors of each annual estimate, not measures of variability across individual participants. None of the corresponding sex-by-year interaction tests reached statistical significance (Supplementary Table S1).

Beyond the pooled 1–11-year comparison in Table 1, Figure 2 presents year-to-year changes (2017–2021) in the seven food groups and macronutrients with a significant overall sex difference (meat, legumes, cola, carbohydrate, protein, animal protein, and fat), restricted to the 6–11-year band—the age range immediately preceding the typical clinical threshold for PP diagnosis. Within this band, boys’ intake remained significantly higher than girls’ for six of the seven variables when tested specifically within this age band (meat, cola, carbohydrate, protein, animal protein, and fat; all *p* = 0.008, Table 1); the exception was legumes, for which the overall sex difference (*p* = 0.047) was not significant within the 6–11-year band alone (*p* = 0.421), reflecting substantial year-to-year fluctuation in this food group in both sexes. Numerically, the sex gap narrowed over 2017–2021 for carbohydrate, protein, animal protein, and fat, and widened for meat and cola despite both rising in absolute terms (full annual values shown in Figure 2); however, as detailed below, none of these year-to-year patterns reached statistical significance, so they are reported only as descriptive observations. However, when these year-to-year patterns were formally tested using a sex-by-year interaction model, none reached statistical significance (all *p* = 0.34–0.81; Supplementary Table S1), reflecting the limited number of annual observations (five years per sex) available for this test. These numerical patterns should therefore be interpreted as descriptive rather than as confirmed statistical trends.

### 3.4. Sex Differences in Food Group and Nutrient Intake

To characterize sex differences in dietary intake, the Mann–Whitney U test was conducted on the average daily intake data of the 12 food groups, treating each annual sex-specific estimate as the unit of comparison (five years per sex). Table 1 presents the results of this analysis. Boys displayed higher intake of carbohydrates, total protein, and animal protein (all *p* = 0.009) than girls. These three differences remained significant, as did cola (*p* = 0.009) after Benjamini–Hochberg correction for the 16 comparisons performed. Fat intake was also higher in boys (*p* = 0.016), though this difference did not survive multiple-testing correction; legumes and meat showed a similar nominal difference (*p* = 0.047 for both) that likewise did not survive correction. In contrast, girls showed a higher intake of seaweed than boys, although this difference was not statistically significant (*p* = 0.203). Given the small effective sample size (five annual estimates per sex), these comparisons should be interpreted as descriptive rather than strongly inferential, and absolute median intake disparities were in all cases modest, with overlapping consumption patterns. When re-tested within the 6–11-year band specifically (Table 1), the sex difference became more consistent for cola, carbohydrate, protein, animal protein, and fat (all *p* = 0.008) and for meat (*p* = 0.008), but remained non-significant for legumes (*p* = 0.421); this age-band-specific comparison is described further in the following section. Because these estimates are not fully independent and are based on only five observations per sex (Section 2.2.1), the resulting *p*-values are necessarily coarse and should be interpreted as descriptive rather than as strong inferential evidence. After Benjamini–Hochberg FDR correction (q = 0.05) across these 16 comparisons, only cola, carbohydrate, protein, and animal protein remained significant; fat (*p* = 0.016) fell just short of the corrected threshold, and legumes and meat (both *p* = 0.047) no longer reached significance (Supplementary Table S3). Notably, meat’s sex difference, though not significant after correction in the pooled comparison, was far stronger and remained significant after FDR correction when re-tested specifically within the 6–11-year band (*p* = 0.008; Table 1), consistent with this study’s broader argument that combining across a wide age range can obscure age-band-specific dietary differences. Because these seven variables were selected based on first-stage statistical significance rather than a priori biological hypotheses (Section 2.2.1), this data-driven selection introduces a selection bias, and the resulting *p*-values—including the year-to-year interaction tests (Figure 2) and the 6–11-year re-test (Table 1)—should be interpreted as exploratory rather than as independent confirmatory evidence.

In the present data, the modest overall sex difference in legume intake (*p* = 0.047, Table 1) was not present within the 6–11-year band specifically (*p* = 0.421) and did not show a significant year-to-year trend (sex-by-year interaction *p* = 0.468; Figure 2).

### 3.5. Dietary Pattern Structure Among Food Groups and Nutrients

To examine the underlying structure of dietary intake, a PCA restricted to the 12 food groups was conducted using the 10 sex-year observations for children aged 6–11 years (2017–2021). The first two principal components accounted for 31.4% and 19.3% of the total variance, respectively (50.7% cumulative). The first component separated boys’ and girls’ dietary profiles, with nearly all food groups—most strongly meat, cola, carbonated drinks, eggs, and other sweetened beverages—loading in the boys’ direction; fruit drinks was the sole exception, loading weakly in the opposite direction, consistent with the general pattern of higher absolute food-group intake among boys described in Table 1. Because this PCA was performed on absolute gram-per-day intake rather than energy-adjusted or intake-density values, PC1 may partly reflect differences in overall food volume and body size between boys and girls rather than purely compositional differences in diet; the individual-level data needed for energy or body-size adjustment were not available to us (Section 4.4). This sex-separation axis was robust to the exclusion of any single survey year in a leave-one-year-out sensitivity analysis (Section 2.2.2). Along this axis, both sexes’ profiles moved toward the origin between 2017 and 2021 (boys’ scores declining from an extreme 2017 value, girls’ scores shifting in the opposite direction), numerically consistent with the narrowing sex gap observed for several macronutrients within the 6–11-year band, although these year-to-year patterns were not statistically significant on their own (Section 3.3). The second component distinguished a dairy/seaweed/beverage-leaning profile (dairy, seaweed, fruit drinks, other sweetened beverages, carbonated drinks, seafood) from a meat/egg/cola-dominant profile (meat, cola, eggs, cider, fermented beverages), independent of overall intake volume; legumes loaded only weakly on this axis, sitting close to the origin rather than aligning clearly with either profile. In contrast to PC1, this secondary axis was comparatively unstable in the same sensitivity analysis, particularly when 2020 was excluded (Section 2.2.2), and should therefore be interpreted with more caution. This secondary axis may partly reflect broader dietary or age-related patterns among Korean children: dairy consumption is often emphasized in early-to-middle childhood nutrition guidance (e.g., school-provided milk programs) to support bone growth, whereas meat, eggs, and cola may be more characteristic of a Westernized, snack-oriented dietary style; this contrast is speculative, however, and was not directly tested in this study.

**Figure 3:**
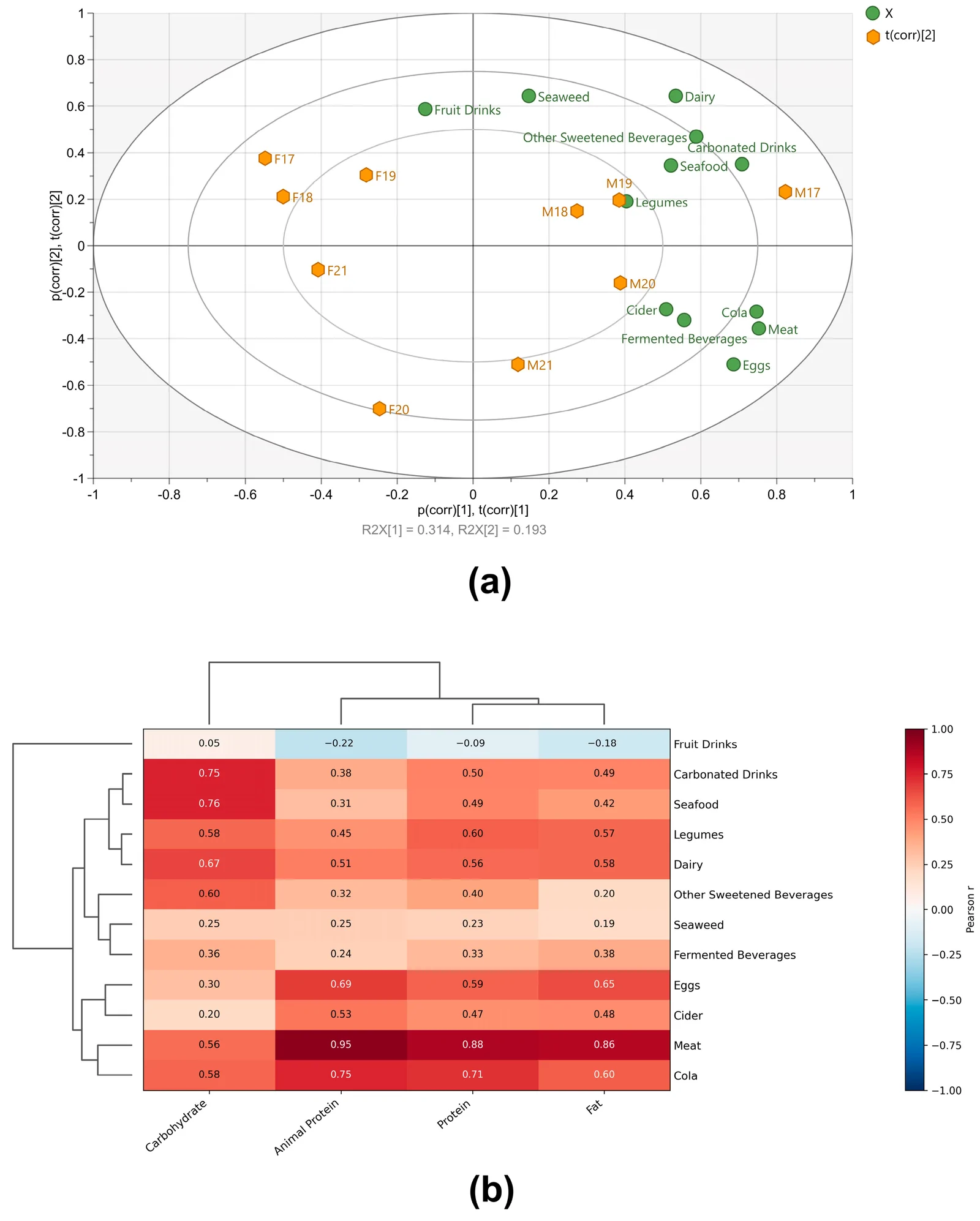
Dietary pattern structure among the 12 KNHANES food groups, children aged 6–11 years (2017–2021; five annual values per sex). (**a**) Principal component analysis biplot restricted to the 12 food groups (macronutrients, disease counts, and population size excluded as input variables); green circles show food-group loadings, points show sex-year scores (M = boys, F = girls; e.g., M17 = boys, 2017). R2X[1] = 31.4%, R2X[2] = 19.3%. (**b**) Hierarchical clustering heatmap of Pearson correlations between each food group and the four macronutrients (carbohydrate, protein, animal protein, fat) across the same five annual values per sex (10 sex-year observations total). Full PC1/PC2 loadings and a leave-one-year-out sensitivity analysis are reported in Supplementary Table S2; given the small number of observations, both panels should be interpreted as exploratory.

Because the four macronutrients are derived in part from the food groups themselves, their relationship to this food-group pattern was examined separately using Pearson correlation with hierarchical clustering (Figure 3b) rather than within the same PCA. This analysis identified three clusters. Meat, cola, eggs, and cider clustered together, each showing moderate-to-very-strong positive correlations with animal protein, protein, and fat (r = 0.47–0.95); meat showed the strongest association (animal protein r = 0.95, protein r = 0.88, fat r = 0.86). This strong meat–animal protein–fat association is expected in part because animal protein and fat are macronutrient components derived directly from meat and other animal-source foods rather than independently measured quantities, so it reflects a partly definitional relationship rather than a novel biological finding; the associations for food groups without such a direct compositional link to the correlated macronutrient (e.g., legumes, dairy) are more informative for this comparison. A second, larger cluster—carbonated drinks, seafood, legumes, dairy, other sweetened beverages, seaweed, and fermented beverages—correlated more moderately with carbohydrate (r = 0.25–0.76) and more weakly with protein, animal protein, and fat (r = 0.19–0.60); legumes and dairy showed the most balanced correlation across all four macronutrients within this cluster (r = 0.45–0.67). Fruit drinks stood apart as the only food group showing near-zero or weakly negative correlations with all four macronutrients (r = −0.22 to 0.05), consistent with its distinct loading on the PCA (Figure 3a). As with the PCA, these clusters are based on only 10 sex-year observations and should be read as a descriptive summary of the correlation structure within this specific dataset rather than as validated, generalizable dietary groupings.

As an exploratory, hypothesis-generating supplementary analysis, we additionally examined within-sex correlations between annual food-group intake and PP care-seeking rate per 100,000 population (2017–2021; *n* = 5 year-points per sex); given the very small number of observations underlying these correlations, full results are reported in the Supplementary Materials (Supplementary Note S1) rather than in the main text, and were not used to prioritize candidate exposures (Section 4.2).

## 4. Discussion

### 4.1. Potential Biological Context for Sex Differences in Pubertal Timing

The overall rise is consistent with prior epidemiological reports of a sex-specific increase in PP-related care in Korea [16,26]. The mechanistic hypotheses discussed below are drawn from the external literature on pubertal biology and metabolism; because the population-level, unlinked design used here does not measure hormonal, metabolic, or psychosocial variables directly, none of these pathways can be tested with the present data, and they should be read as candidate explanations for the patterns described in Section 3, not as findings established by this study.

Sex-specific differences in dietary intake were noted for several food groups and macronutrients, with boys demonstrating higher median intakes for most. These findings indicate that dietary patterns are sex-divergent even in early childhood, although the absolute differences in intake were modest, with substantial overlap between groups. Although high intake of animal protein and fat has been associated with earlier pubertal onset through mechanisms involving insulin-like growth factor 1 secretion and hypothalamic inflammation [27], these pathways are likely influenced by broader metabolic, environmental, and psychosocial factors, and cannot be tested directly with the population-level design used here. Cola intake may reflect a related but distinct pathway: sugar-sweetened beverage intake is linked to pediatric obesity, and childhood obesity/adiposity is among the most consistently reported risk factors for earlier pubertal onset via leptin-mediated activation of the hypothalamic–pituitary–gonadal axis [28] (see also [14,15]), raising cola as a possible contributing factor to this pathway. Notably, national childhood/adolescent obesity prevalence in Korea has risen more steeply among boys than girls over the past decade (10.4% to 25.9% vs. 8.8% to 12.3%, 2012–2021 [29])—a sex divergence in the same direction as the disproportionate rise in boys’ cola intake observed here (Figure 2)—suggesting cola/sugar-sweetened beverage intake may relate to PP risk through a general, sex-nonspecific obesity pathway rather than the female-predominant convergence pattern seen for other food groups; this parallel is descriptive only and not evidence of a causal link.

The pronounced sex disparity in PP care-seeking (Figure 1) may be partly attributable to sex-specific biological sensitivity during prepubescence rather than to dietary volume alone. While boys generally exhibit higher absolute nutrient intake, girls tend to have a greater proportion of body fat during childhood, and measures of adiposity are more closely linked to pubertal timing than body mass index (BMI) alone [30,31]. Adipose tissue regulates puberty via adipokines, particularly leptin, a key metabolic signal for activating the HPG axis [28], and circulating leptin levels are higher in girls than in boys at similar developmental stages [32,33]. Therefore, even moderate caloric or fat intake in girls may be sufficient to reach the metabolic threshold required for HPG axis activation—an intrinsic biological sensitivity that, rather than dietary volume, may partly explain likely underlies much of the sex disparity described in Figure 1. This leptin-mediated pathway is specific to central precocious puberty; because the HIRA-derived counts in Figure 1 do not distinguish central from peripheral cases (Section 2.1.1), this mechanism may not account for the full sex disparity observed here.

Together, these patterns are consistent with the literature-derived hypothesis that cola’s disproportionate rise among boys reflects a shared, sugar/caloric-intake-mediated obesity pathway distinct from the isoflavone- and animal-protein-mediated pathways proposed for legumes and meat, respectively (see Section 4.2).

### 4.2. Legumes, Seaweed, and Other Candidate Dietary Exposures

Among the dietary variables examined, legumes deserve special attention, though the present data urge caution rather than emphasis. Isoflavones found in legumes, especially soy-based foods, are structurally similar to 17β-estradiol and can bind to estrogen receptors [34]. Previous studies have reported higher circulating isoflavone levels in girls with precocious puberty than in healthy controls [35], although a meta-analysis found no significant association between early childhood nutrient intake and PP incidence overall [36]. These findings neither support nor rule out a role for legume/isoflavone intake and should not be over-interpreted as evidence of convergence. In the dietary pattern analysis (Section 3.5), legumes clustered with dairy in the food-group–macronutrient correlation structure (Figure 3b) but loaded only weakly on the PCA’s secondary axis (Figure 3a), sitting near the origin rather than clearly opposing a meat-dominant profile—reflecting co-variation among food groups rather than a direct relationship with PP-care-seeking rates, which was not included in either analysis. This secondary-axis structure was also comparatively unstable in the leave-one-year-out sensitivity analysis (Section 2.2.2), further supporting a cautious, hypothesis-generating interpretation of this pattern rather than treating it as an established dietary grouping. Seaweed’s inclusion as a candidate exposure also has a plausible, though indirect and untested, biological rationale: several seaweed species are exceptionally rich in iodine [37], and iodine-driven thyroid dysfunction is a recognized, reversible cause of precocious puberty through TSH’s cross-reactivity with the FSH receptor (Van Wyk-Grumbach syndrome) [38]; however, no study to our knowledge has directly examined seaweed or iodine intake in relation to pubertal timing, so this pathway remains considerably more speculative than the isoflavone-mediated mechanism proposed for legumes. Population-level legume intake, as measured here, is also an imperfect proxy for isoflavone exposure specifically: in a study of Korean children aged 8–11 years, mean daily isoflavone intake was modest (8.3 mg in boys, 7.2 mg in girls), with more than 70% of children below 10 mg/day, and tofu and soybean-based foods—rather than legumes as a whole food-group total—were the main contributors [39]. Because our legume variable aggregates all legume-derived foods rather than isolating soy-isoflavone content specifically, it may not meaningfully track isoflavone exposure even where legume intake itself is well measured.

Because both dietary intake and PP care-seeking rose over the same five-year window, the exploratory within-sex correlations reported in the Supplementary Materials (Supplementary Note S1) are highly susceptible to confounding by a shared secular trend and cannot be distinguished from it with only five annual observations per sex. These associations should not be interpreted as evidence of an individual-level dietary effect; they were not used as the primary basis for prioritizing candidate exposures. Where animal protein and legumes are discussed as directions for future individual-level research, that motivation rests primarily on biological plausibility rather than on these exploratory correlations; seaweed’s inclusion likewise has a plausible biological rationale (iodine-thyroid effects, discussed above), though a considerably more indirect and untested one than the mechanisms proposed for legumes or animal protein/fat, and should be interpreted with correspondingly greater caution.

### 4.3. Comparison with Prior Studies and Synthesis

Collectively, the univariate and multivariate analyses indicate significant sex-specific differences and trends in food group intake among Korean children over 2017–2021. Restricting part of this analysis to the 6–11-year band—rather than pooling across ages 1–11 as in most prior surveillance reports—revealed that some sex differences (meat, cola, and the four macronutrients) are, if anything, more consistent in this age band, while others (legumes) are not, illustrating that age-pooled dietary summaries can both mask and manufacture apparent sex-specific patterns depending on the food group. These results align with recent population-based studies in Chinese children, which reported associations between traditional dietary patterns and early or precocious puberty while also emphasizing the constraints of cross-sectional designs and potential confounding factors [40].

### 4.4. Limitations

In this study, our analysis of KNHANES itself relies on publicly released annual sex-stratified aggregate estimates rather than individual-level microdata; we did not have access to a data-use agreement permitting reanalysis with individual respondent records, sampling weights, strata, or primary sampling units, which would allow a more statistically robust survey-design-based comparison of boys’ and girls’ intake. We identify such a reanalysis as an important direction for future work. Relatedly, because our dietary comparisons and PCA are based on absolute gram-per-day intake, they cannot distinguish true differences in diet composition from differences in overall food volume, energy intake, body size, physical activity, or growth stage between boys and girls; energy-adjusted or intake-density analyses, which would help disentangle these possibilities, were not feasible without individual-level data. These findings should be interpreted considering several constraints inherent to population-level design. Because neither KNHANES (a general nutrition survey with no PP diagnosis variable) nor HIRA (aggregate care-seeking counts with no dietary data) permits individual-level linkage, this study describes population-level dietary trends rather than individual dietary intake, and no causal, individual-level, or predictive risk claims can be drawn from the analyses presented here. This aggregation extends to the statistical comparisons themselves: because publicly available KNHANES summary statistics are reported at the level of age-band–year cells rather than individual respondents, the overall food-group comparisons in Table 1 are calculated from only five annual values per sex, and the 6–11-year-band comparisons likewise from five annual values for that band alone—both should be read as comparisons of population subgroup estimates rather than individual-level observations. The HIRA-derived rates in Figure 1 add a further layer of imprecision, as they reflect all care coded under disease classification E301, including diagnostic work-up and follow-up visits, and are therefore not directly comparable in absolute magnitude to previously published incidence estimates for confirmed PP diagnoses. This same small-sample constraint limits the exploratory within-sex correlations, the year-to-year trend comparisons in Figure 2, and the sex-by-year interaction tests, all of which draw on only five annual observations per sex; the interaction tests in particular were underpowered to detect the numerical narrowing or widening patterns described in Section 3.3 (all *p* > 0.3) and remain vulnerable to confounding by shared secular trends such as rising childhood obesity, pandemic-era lifestyle change, and shifts in diagnostic ascertainment, so they should be read as hypothesis-generating only. Addressing these constraints will require linked national datasets that connect individual dietary or feeding-behavior data with confirmed PP diagnosis—for example, national health screening programs linked to insurance-based diagnosis, as demonstrated by Lee et al. [4] for early-life feeding factors and PP—or case–control studies with individual-level dietary assessment [18,19]. Such individual-level data would also be a prerequisite for any predictive or regression-based risk model for PP, which the aggregate, population-level design used here cannot support.

## 5. Conclusions

In conclusion, Korean children’s dietary intake showed sex differences that, while modest in absolute magnitude and overlapping between sexes, were, for most food groups and macronutrients examined, at least as consistent within the 6–11-year band—the age range immediately preceding the typical clinical threshold for PP diagnosis—as in the pooled 1–11-year population, coinciding with a period of rising national PP care-seeking rates. These comparisons rest on only five annual observations per sex and are descriptive, not confirmatory. Numerical year-to-year narrowing (carbohydrate, protein, animal protein, fat) and widening (meat, cola) patterns in this age band did not reach statistical significance and should be treated as descriptive rather than confirmed trends. Population-level dietary intake volume does not, on its own, explain the sex disparity in PP care-seeking rates, and this study does not claim to establish such an explanation. Instead, these descriptive findings identify animal protein and fat as candidate dietary exposures warranting investigation using individual-level study designs, motivated primarily by their biological plausibility in the existing literature; legumes and seaweed remain of more tentative interest, supported by plausible but indirect biological rationales—isoflavone-mediated estrogen receptor binding for legumes, iodine-driven thyroid effects for seaweed—rather than by consistent direct evidence, and should be interpreted with correspondingly greater caution. These results underscore the need for caution against oversimplified dietary narratives, and support continued nutritional surveillance of Korean children alongside future case–control research linking individual dietary data to confirmed PP diagnosis. Specific priorities for this future work include longitudinal cohort studies that track dietary intake and pubertal timing within the same children over time, incorporation of hormonal or metabolic biomarkers (e.g., leptin, insulin-like growth factor 1) alongside dietary assessment, and individual-level linkage studies connecting dietary records directly to confirmed PP diagnosis; together, these designs would be needed to establish whether the candidate exposures identified here play a genuine role in the sex disparity described.

## Figures and Tables

**Table 1:** The comparison analysis of daily intake data of 12 food groups from girls and boys using the Mann–Whitney U test.

Groups	Female Median (IQR ^a^)	Male Median (IQR)	*p*-Value ^b^	*p*-Value (Ages 6–11 Only)
**Foods**				
Legumes	19.20 (14.90–32.70)	27.66 (20.40–34.00)	0.047 *^†^	0.421
Seaweed	2.70 (1.80–3.40)	2.32 (2.20–2.70)	0.203	—
Meat	65.18 (54.70–75.00)	78.10 (70.80–84.10)	0.047 *^†^	0.008 *
Eggs	27.10 (20.80–32.30)	31.96 (28.80–38.70)	0.116	—
Dairy	221.26 (201.90–240.30)	230.92 (199.70–253.70)	0.465	—
Fermented Beverages	4.08 (2.10–7.30)	7.44 (3.10–10.50)	0.076	—
Fruit Drinks	29.50 (21.20–35.70)	35.62 (22.70–50.60)	0.347	—
Other Sweetened Beverages	11.56 (9.20–16.00)	14.68 (11.20–21.40)	0.175	—
Carbonated Drinks	4.10 (1.40–5.80)	5.72 (2.10–8.40)	0.142	—
Cider	4.10 (1.50–7.70)	6.40 (3.70–9.00)	0.117	—
Cola	8.80 (6.50–9.90)	16.24 (13.50–20.20)	0.009 *	0.008 *
Seafood	20.92 (18.40–22.90)	22.54 (18.70–24.80)	0.251	—
**Macronutrients**				
Carbohydrates	203.62 (199.10–208.50)	225.52 (218.50–239.10)	0.009 *	0.008 *
Protein	46.96 (44.50–49.80)	53.04 (52.00–54.70)	0.009 *	0.008 *
Animal Protein	27.40 (25.30–28.70)	31.14 (30.60–31.80)	0.009 *	0.008 *
Fat	37.28 (34.50–40.30)	41.30 (38.80–42.80)	0.016 *^†^	0.008 *

Data are presented as median and interquartile range (IQR) with grams per day. Each median (IQR) is computed across five annual sex-specific aggregate estimates (2017–2021), not across individual KNHANES participants. ^a^ IQR, interquartile range. ^b^ Statistical significance was determined using the Mann–Whitney U test to compare dietary intake between girls and boys (* *p* < 0.05). ^†^ Not significant after Benjamini–Hochberg false discovery rate (FDR) correction (q = 0.05) across the 16 pooled comparisons; see Section 2.2.1 and Supplementary Table S3.

## Data Availability

The KNHANES data analyzed in this study are publicly available from the Korea Disease Control and Prevention Agency (https://knhanes.kdca.go.kr (accessed on 12 May 2024). The HIRA-derived precocious puberty care-seeking counts are available from the Health Insurance Review and Assessment Service upon reasonable request, subject to institutional data-sharing agreements. The derived summary datasets supporting the findings of this study are available from the corresponding author upon reasonable request.

## Supplementary Materials

The following supporting information can be downloaded at: https://www.mdpi.com/article/10.3390/nu18203445/s1, Supplementary Table S1: Sex-by-year interaction test (weighted least squares, weighted by inverse variance of the annual survey estimate) for year-to-year change in intake within the 6–11-year band, 2017–2021, for the seven food groups/macronutrients; Supplementary Table S2: Principal component (PC1, PC2) loadings for the 12 KNHANES food groups from the exploratory PCA restricted to children aged 6–11 years (2017–2021; 10 sex-year observations; R2X[1] = 31.4%, R2X[2] = 19.3%), reported in response to reviewer requests for full loading disclosure given that this PCA uses more variables (12) than observations (10). Sign convention: positive PC1 corresponds to the boys’ direction; Supplementary Table S3: Benjamini-Hochberg false discovery rate (FDR) correction (q = 0.05) applied to the Mann-Whitney U test *p*-values reported in Table 1, in response to reviewer concerns about multiple-testing correction across the 16 comparisons in the overall (ages 1–11 years) screen and the 7 comparisons re-tested within the 6–11-year band; Supplementary Note S1: Exploratory correlations between dietary intake and PP care-seeking rate.

## Abbreviations

The following abbreviations are used in this manuscript:

BMI	Body mass index
HIRA	Health Insurance Review and Assessment Service
HPG	Hypothalamic–pituitary–gonadal
IQR	Interquartile range
KDCA	Korea Disease Control and Prevention Agency
KNHANES	Korea National Health and Nutrition Examination Survey
MOE	Ministry of Education
MSIT	Ministry of Science and ICT
NRF	National Research Foundation of Korea
PCA	Principal component analysis
PP	Precocious puberty
RISE	Regional Innovation System & Education
