# Supplementary material for: Five-Year Trends in Dietary Intake by Gender Among Korean Preschoolers (2017–2021): Nationwide Survey-Based Surveillance Amid Rising Demand for Medical Care for Precocious Puberty
Source: Nutrients. 2026 Oct 20;18(20):3445. doi: 10.3390/nu18203445 (PMC13708578; doi:10.3390/nu18203445)
Supplement: Supplementary file 1 [file nutrients-18-03445-s001.zip › nutrients-4528860-supplementary.pdf]

Supplementary Materials

Table S1. Sex-by-year interaction test (weighted least squares, weighted by inverse variance of the annual survey estimate) for year-to-year change in intake within the 6-11-year band, 2017-2021, for the seven food groups/macronutrients.

Food group / nutrient	Interaction β	p-value	Numerical direction
**Legumes**	−2.415	0.468	Widening
**Meat**	−0.464	0.808	Widening
**Cola**	−1.907	0.400	Widening
**Carbohydrate**	1.792	0.613	Narrowing
**Protein**	0.638	0.627	Narrowing
**Animal Protein**	0.436	0.575	Narrowing
**Fat**	1.174	0.337	Narrowing

A positive interaction coefficient indicates a numerically narrowing sex gap over time (girls' slope increasing faster, relative to boys, than boys' own slope); a negative coefficient indicates a numerically widening gap. None reached statistical significance.

Table S2. Principal component (PC1, PC2) loadings for the 12 KNHANES food groups from the exploratory PCA restricted to children aged 6-11 years (2017-2021; 10 sex-year observations; R2X[1] = 31.4%, R2X[2] = 19.3%), reported in response to reviewer requests for full loading disclosure given that this PCA uses more variables (12) than observations (10). Sign convention: positive PC1 corresponds to the boys' direction.

Food group	PC1	PC2
Fruit Drinks	-0.065	+0.385
Seaweed	+0.076	+0.422
Legumes	+0.208	+0.125
Cider	+0.262	-0.178
Seafood	+0.268	+0.227
Dairy	+0.275	+0.425
Fermented Beverages	+0.286	-0.209
Other Sweetened Beverages	+0.304	+0.308
Eggs	+0.353	-0.335
Carbonated Drinks	+0.366	+0.229
Cola	+0.384	-0.187
Meat	+0.388	-0.235

A leave-one-year-out sensitivity analysis (excluding one survey year at a time, n=8 remaining) showed PC1 loadings were highly stable (correlation to the full-model loadings above: r=0.61-0.97 across the five iterations), whereas PC2 loadings were less stable (r=0.33-0.95), particularly when 2020 was excluded (r=0.33); see Section 2.2.2 and Section 3.5 of the main text.

Table S3. Benjamini-Hochberg false discovery rate (FDR) correction (q = 0.05) applied to the Mann-Whitney U test p-values reported in Table 1, in response to reviewer concerns about multiple-testing correction across the 16 comparisons in the overall (ages 1-11 years) screen and the 7 comparisons re-tested within the 6-11-year band.

(a) Overall (pooled, ages 1-11 years) comparison, m = 16.

Food group / nutrient	Raw p	Rank	BH critical value (rank/m x q)	BH-adjusted p	Significant after FDR
Cola	0.009	1	0.00313	0.0360	Yes
Carbohydrate	0.009	2	0.00625	0.0360	Yes
Protein	0.009	3	0.00938	0.0360	Yes
Animal Protein	0.009	4	0.01250	0.0360	Yes
Fat	0.016	5	0.01562	0.0512	No
Legumes	0.047	6	0.01875	0.1074	No
Meat	0.047	7	0.02188	0.1074	No
Fermented Beverages	0.076	8	0.02500	0.1520	No
Eggs	0.116	9	0.02813	0.1872	No
Cider	0.117	10	0.03125	0.1872	No
Carbonated Drinks	0.142	11	0.03438	0.2065	No
Other Sweetened Beverages	0.175	12	0.03750	0.2333	No
Seaweed	0.203	13	0.04063	0.2498	No
Seafood	0.251	14	0.04375	0.2869	No
Fruit Drinks	0.347	15	0.04688	0.3701	No
Dairy	0.465	16	0.05000	0.4650	No

(b) Re-test within the 6-11-year band, m = 7.

Food group / nutrient	Raw p	Rank	BH critical value (rank/m x q)	BH-adjusted p	Significant after FDR

Meat	0.008	1	0.00714	0.0093	Yes
Cola	0.008	2	0.01429	0.0093	Yes
Carbohydrate	0.008	3	0.02143	0.0093	Yes
Protein	0.008	4	0.02857	0.0093	Yes
Animal Protein	0.008	5	0.03571	0.0093	Yes
Fat	0.008	6	0.04286	0.0093	Yes
Legumes	0.421	7	0.05000	0.4210	No

BH-adjusted p (the Benjamini-Hochberg-corrected p-value, sometimes called the q-value) is the smallest FDR level at which a given comparison would be called significant; a comparison is significant at q = 0.05 when its BH-adjusted p is <= 0.05, equivalently when its raw p is <= its own critical value and no higher-ranked (larger-p) comparison also satisfies this within the same procedure. See Section 2.2.1 and Section 3.4 of the main text.

Supplementary Note S1. Exploratory correlations between dietary intake and PP care-seeking rate.

As an exploratory, hypothesis-generating supplementary analysis, we examined within-sex correlations between annual food-group intake and PP care-seeking rate per 100,000 population (2017-2021; n = 5 year-points per sex). Seaweed intake showed a weak positive within-sex correlation with care-seeking rate in both boys (r = 0.26) and girls (r = 0.19), while several other food groups (e.g., animal protein, fat, meat) showed stronger within-sex correlations in girls (r = 0.70-0.91). Because both dietary intake and PP care-seeking rose over the same five-year window, these correlations are highly susceptible to confounding by a shared secular trend and cannot be distinguished from it with only five annual observations per sex; they should not be interpreted as evidence of an individual-level dietary effect, and were not used as the primary basis for prioritizing candidate exposures in the Discussion (Section 4.2).
